# Systems epidemiology of metabolomics measures reveals new relationships between lipoproteins and other small molecules

**DOI:** 10.1007/s11306-021-01856-6

**Published:** 2021-12-16

**Authors:** Fotios Drenos

**Affiliations:** 1 Department of Life Sciences, College of Health, Medicine and Life Sciences, Brunel University London, Uxbridge, UB8 3PH UK; 2 Institute of Cardiovascular Sciences, UCL, London, WC1E 6JF UK

**Keywords:** ALSPAC, Metabolomics, Genetic correlation, Causality, Mendelian randomization, Systems epidemiology

## Abstract

**Introduction:**

The study of lipoprotein metabolism at the population level can provide valuable information for the organisation of lipoprotein related processes in the body. To use this information towards interventional hypotheses generation and testing, we need to be able to identify the mechanistic connections among the large number of observed correlations between the measured components of the system.

**Objectives:**

To use population level metabolomics information to gain insight on their biochemical networks and metabolism.

**Methods:**

Genetic and metabolomics information for 230 metabolic measures, predominately lipoprotein related, from a targeted nuclear magnetic resonance approach, in two samples of an established European cohort, totalling more than 9400 individuals analysed using phenotypic and genetic correlations, as well as Mendelian Randomisation.

**Results:**

More than 20,500 phenotypic correlations were identified in the data, with almost 2000 also showing evidence of strong genetic correlation. Mendelian randomisation, provided evidence for a causal effect between 9496 pairs of metabolic measures, mainly between lipoprotein traits. The results provide insights on the organisation of lipoproteins in three distinct classes, the heterogeneity between HDL particles, and the association, or lack of, between CLA, glycolysis markers, such as glucose and citrate, and glycoproteins with lipids subfractions. Two examples for the use of the approach in systems biology of lipoproteins are presented.

**Conclusions:**

Genetic variation can be used to infer the underlying mechanisms for the associations between lipoproteins for hypothesis generation and confirmation, and, together with biological information, to map complex biological processes.

**Supplementary Information:**

The online version contains supplementary material available at 10.1007/s11306-021-01856-6.

## Introduction

Metabolomics is the study of the quantitative complement of small molecules in biological systems. The metabolic measures obtained are mostly organic compounds involved in the biochemical reactions of the organism and represent the final stage of the flow of information from the genome to the biological phenotype (Dunn et al., 2011a). One of the main characteristics of metabolomics approaches is their ability to obtain multiple measures from a biological pathway, representing its intermediate steps and chemical compounds involved. As expected, these measures are usually tightly correlated with each other and as metabolic pathways intersect, these correlations can be extensive among metabolomics data (Camacho et al., 2005). For lipoproteins measures the interactions of the particles during lipids metabolism give rise to a large number of correlations that complicate our understanding of their impact on health (Holmes et al., 2015). Understanding the nature of these correlations and the flow of information between them leading to the observed phenotype is a challenging problem and the focus of systems studies (Dunn et al., 2011b). Distinguishing between lipoprotein measures belonging in the same process and those that are correlated due to other reasons has implication on our understanding of the underlying causes for disease and the identification of relevant interventional strategies (Steuer, 2006).

In contrast to phenotypic correlations between biological measures, genetic correlations suggest the presence of a partially shared underlying genetic mechanism (Bulik-Sullivan et al., 2015a; Lee et al., 2012). Genetically correlated metabolic measures can be considered as taking part in the same biological process, though each one might also affect other pathways and processes in the system. In addition to the existence of common mechanisms between metabolic measures, we are also interested in the flow of information between them. Mendelian randomization (MR) is a popular method able to assess the direction of effect between two correlated traits (Davey Smith & Hemani, 2014). In this case, genetic polymorphisms are used as instruments to estimate the causal effect between the two measures and identify pairs of metabolic traits that are part of the same chain of effects from those correlated through other mechanisms.

Here, using a well characterised European population cohort, of children and their mothers, and metabolomics measures from a targeted NMR approach focusing mainly on lipoproteins, fatty acids, and amino acids, I aimed to elucidate the relationships between the available metabolic measures. Understanding of the relationships between the metabolic measures can then be used to test hypotheses.

## Methods

### Study population

The Avon Longitudinal Study of Parents and Children (ALSPAC) is a population based, prospective birth cohort (www.bris.ac.uk/alspac). The study recruited 14,541 pregnancies and has since followed participants in a number of phases during development and maturity. Full details of the study have been published previously (Boyd et al., 2013; Fraser et al., 2013). Here we use the unrelated offspring of this study at age 7, as the discovery sample, and mothers from the first focus on mothers sample collection, as replication. The study website contains details of all the data that is available through a fully searchable data dictionary http://www.bris.ac.uk/alspac/researchers/data-access/data-dictionary/. Ethical approval for the study was obtained from the ALSPAC Ethics and Law Committee and from the UK NHS National Health Service Local Research Ethics Committees. Participants have provided informed consent for the use of the data.

### Serum NMR metabolomics

A high-throughput serum nuclear magnetic resonance (NMR) metabolomics platform was used to quantify 230 metabolic measures representing a broad molecular signature of systemic metabolism (Soininen et al., 2015). The measured set covers multiple metabolic pathways, including lipoprotein lipids and subclasses, fatty acids and fatty acid composition, as well as amino acids and glycolysis precursors. This applied NMR-based metabolic profiling platform has recently been used in various epidemiological and genetic studies (Beaney et al., 2016; Drenos et al., 2016; Würtz et al., 2016a). Applications of this high-throughput metabolomics platform has been reviewed (Soininen et al., 2015) and details of the experimentation have been described elsewhere (Soininen et al., 2009). Previous work (Würtz et al., 2017) has shown excellent correlation between the NMR determined measures and their respective clinically assessed values.

For 5645 of the ALSPAC young participants, 48.5% females, metabolic measures were obtained from serum under non-fasting conditions. For 4530 ALSPAC mothers, at a median age of 48 years (IQR 45-51), the measures were obtained from overnight, or at least 6 h, fasted samples.

### Genotyping

The ALSPAC children were genotyped using the Illumina HumanHap610 array. The ALSAPC mothers were genotyped with the Illumina HumanHap550 array. Standard metrics were employed to assess the quality of these data: individual call rate > 97%; heterozygosity threshold: 0.34; minor allele frequency of < 0.005%; SNP call rate of > 97%; and Hardy–Weinberg equilibrium (HWE) (*p* < 5 × 10^−7^) (Bønnelykke et al., 2013).

### Statistical analysis

The metabolomics measures were transformed using a rank-based inverse normal transformation (Blom, 1958). The SNPs effects on the metabolites were obtained through PLINK (Purcell et al., 2007) using a linear model adjusted for age or sex, as appropriate. Independent SNPs were obtained using a pairwise linkage disequilibrium of r^2^ < 0.01 per chromosome through PLINK. When a pair of siblings was present in the data, one of them was randomly removed from the sample. The phenotypic correlation between metabolic measures was represented by the Pearson’s correlation coefficient of their transformed values. Pearson’s correlation coefficients and their respective p-values were estimated through the cor.test in R (Team, 2008). The genetic correlation was estimated through (1) the LD score regression (Bulik-Sullivan et al*.*, 2015b), as found in LD Hub (Zheng et al., 2017), for measures with previous genome wide association study (GWAS) results available, (2) Bivariate REML on individual level data, for measures with no published GWAS (Lee et al., 2012), and (3) approximated by the inverse variance weighted regression between the normalised beta coefficients of the independent SNP-trait associations, for a small number of pairs where both LD score and GCTA algorithms failed to converge. Principal component analysis (PCA) was performed through the caret package in R (Kuhn, 2008). Only independent SNPs with an effect > 3 standard deviations from zero were used for Mendelian randomisation analysis. We used individual SNPs estimates in the weighted inverse variance and MR-Egger methods, as described elsewhere (Bowden et al., 2015), to obtain the causal effects between the traits and test for pleiotropy. The tree and network of lipoproteins characteristics presented were constructed through igraph in R (Csardi & Nepusz, 2006). The rooted tree was based on an algorithm starting with the largest particle and selecting the next particle based on the -log of the association p-value. P-values higher than the threshold were considered as equal to 1. All plots were constructed in R using either the corrplot (Simko, 2016) or ggplot2 packages (Wickham, 2016).

## Results

### Samples characteristics

In the Avon Longitudinal Study of Parents and Children (ALSPAC), excluding all subjects with missing values, resulted in 5353 unrelated children at age 7 and 4120 mothers with available metabolomics measurements. In the ALSPAC offspring, principal component analysis showed that 44 principal components (PCs) accounted for 0.99 of the metabolic measures variance. The corresponding figure in the mothers sample was 0.98. Based on this, our Bonferroni adjusted p-value threshold for the pairwise correlations was set at 5.28 × 10^–5^ for correlation tests and 2.64 × 10^–5^ for regression based tests. In total 465,740 genotyped SNPs passed the quality control criteria in the two samples, with 12,516 SNPs found to be independent (LD r^2^ < 0.01) and used to estimate the correlation of SNP effects between the traits and in selecting MR instruments.

### Phenotypic correlations

Of the 26,335 Pearson’s correlation coefficients tested between the metabolic measures, 23,032 showed evidence of phenotypic association. A table showing all correlation coefficients and their respective *p*-values can be found in the Supplementary material (Table S1) and plotted in Fig. S1. When the lipoprotein measures were ordered by size, as the current understanding links size to function, four clusters of high correlation were evident in their concentration measures, two major and two minor. The first major cluster included Chylomicrons and extra-large very low density lipoprotein (VLDL) and extended to very-large, large, medium and small VLDL particles. The second major cluster included very-small VLDL, intermediate density lipoprotein (IDL) and the various sizes of low density lipoprotein (LDL). The majority of measures were associated across the two clusters except triglycerides and free cholesterol measures. Both of the minor clusters of strong correlations were in the high-density lipoprotein (HDL) measures, with one cluster including particles of very-large and large size and the second measures of very-small HDL. Medium size HDL measures were associated with both. Again, cross cluster correlations were present throughout. The other prominent feature of the phenotypic correlations matrix was the complex correlations pattern between glycolysis, amino acids, ketone bodies, fluid balance, and inflammation markers with lipids, although most were consistently associated with the larger VLDL particles measures. Results from the mothers were similar, replicating 20,758 of the associations, and showed the same major features. The results can be seen in Table S2 and Fig. S2.

### Genetic correlations

Of the 26,335 possible pairs of metabolic measures, 5050 were tested through LD score regression (Bulik-Sullivan et al*.*, 2015a) in LD Hub (Zheng et al., 2017) from external data (Kettunen et al., 2016) with 1551 showing evidence of genetic correlation, 24,484 were estimated using bivariate REML (Lee et al., 2012) in the children sample with 2330 showing evidence of genetic correlation, while 24,121 had evidence of correlation when the beta coefficients of the independent SNPs were considered. Tables S3 and Fig. S3, show the correlation coefficients and their p-values obtained for all pairs of measures. The Pearson correlation between the estimates of the three methods were: 0.753 (CIs 0.740–0.766) between LD score and bivariate REML, 0.827 (CIs 0.818–0.835) between LD score and the correlation of SNP effects and 0.836 (CIs 0.832–0.839) for bivariate REML and the correlation of SNP effects. The main clusters of high correlations present in the phenotypic level were also evident for the genetic correlations, but in this case, the pairwise associations tended to be confined mostly within the observed clusters. The majority of VLDL measures were associated with each other. IDL and LDL measures, formed another cluster of genetic correlations and they were also correlated to some of the medium and smaller VLDL measures. Very large and large HDL measures had evidence of genetic correlations between them, which were less pronounced for medium HDL and mostly absent for small HDL measures. Measures of large HDL showed evidence of correlation with VLDL measures. Fatty acids were correlated to the measures of IDL, LDL and the larger HDL, while the unsaturated fatty acids were also correlated with VLDL measures. Finally, both isoleucine and glycoproteins acetyls had evidence of correlation with VLDL measures. The main patterns of correlation were replicated in the mothers sample, where bivariate REML identified 5040 pairwise correlations while 24,459 pairs of metabolites had correlated SNP effects (Table S4 and Fig. S4).

**Fig. 1:**
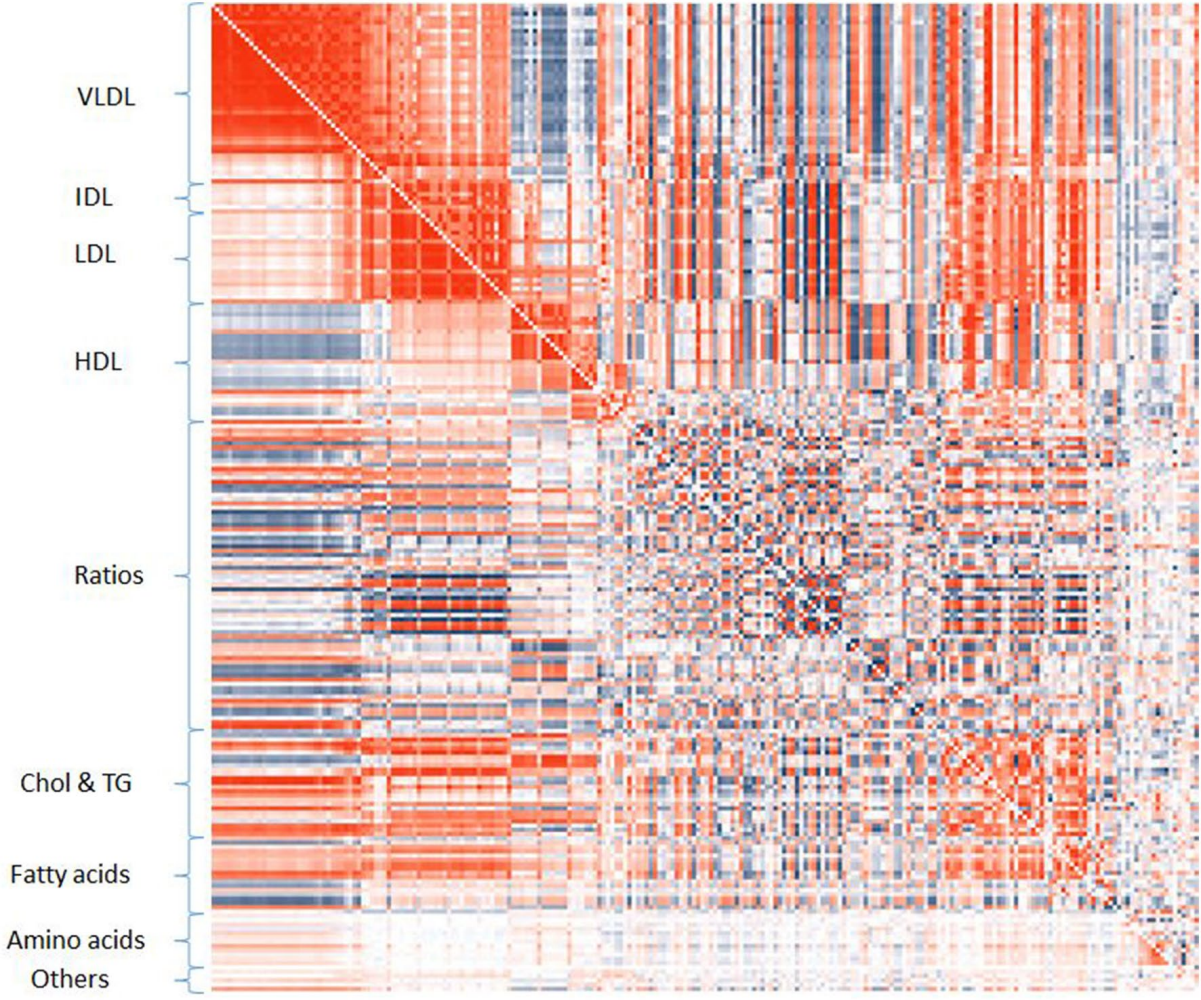
Correlations between 230 metabolites measured through a targeted metabolomics NMR platform. Red for positive correlation and blue for negative. The lower left part of the square shows the correlation between the levels of the metabolic measures. The upper right part shows the correlations of their genetic effects. High degree of similarity is evident in the two triangles in terms of the sign and level of correlation. Only some of these correlations are statistically significant for both levels of correlation

In general, the magnitudes of phenotypic correlations and genetic correlations between the measures had similar patterns (Fig. 1), though their statistical significance evidence differed. There were 3579 correlation pairs where both phenotypic and genetic correlations were evident and 19,543 pairs of measures that were correlated in the phenotypic level but not the genetic level. In the mothers sample, 1966 of the 3579 and 15,692 of the 19,543 relationships were replicated. These 1966 pairs can be found in Table S5.

### Mendelian randomisation

**Fig. 2:**
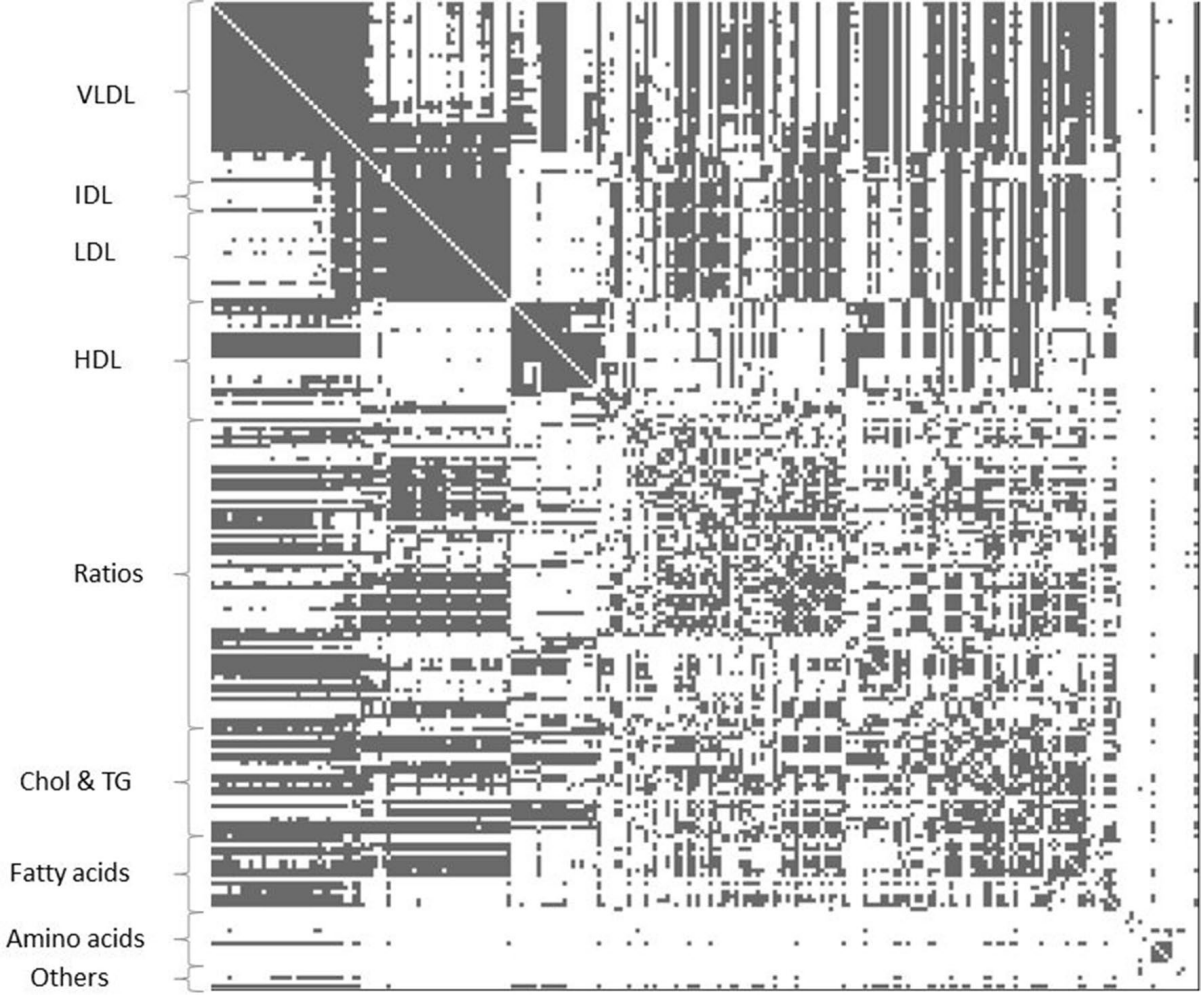
Evidence of MR effects of the row metabolic measure on the column metabolic measure obtained from a targeted metabolomics NMR platform. Grey for a causal association, white for associations not reaching the pre-specified p-value threshold

To better assess the nature of correlations seen between the metabolomics measures, we performed MR analysis for all pairs in both directions. The number of SNPs used for each of the metabolic measures can be seen in Table S6. Of the 52,670 associations tested 26,909 had evidence of a causal effect. The full list of results can be seen in Tables S7 and their respective p-values in Table S8. From the 26,909 relationships observed in the offspring data, 20,663 were replicated in the mother’s sample. Of these, 9297 were bidirectional associations, out of the 12,188 observed in the discovery sample (Table S9), and 199 one-directional causal effects, from the 2533 observed initially (Table S10), with the rest being bidirectional associations replicated in one direction. Figure 2, summarises all the replicated p-values. The three major clusters evident in the genetic correlation results around VLDL, IDL and LDL, and larger HDL particles were again visible as bidirectional associations. The VLDL cluster included measures of the largest lipoprotein particles, such as chylomicrons and very large VLDL particles, to smaller VLDL. The small VLDL measures were associated with both the VLDL cluster and the remnant and LDL cluster of associations. The VLDL cluster was also bidirectionally associated with triglycerides levels in remnant particles and medium and small HDL, but not with triglycerides in large and very large HDL. Phospholipids on LDL, and the large and very large HDL subclasses were associated with the VLDL cluster. Large HDL particle measures also had multiple associations with the VLDL cluster for total, esterified and free cholesterol measures. The remnant lipoprotein and LDL measures cluster were associated with overall esterified and free cholesterol as well as total and esterified cholesterol in small HDL particles. The MR results support a clear distinction between the larger HDL lipoproteins (extra-large and large) and small HDL. Medium HDL measures are more similar to the larger HDL measures, though they also have overlapping effects with total lipids and triglycerides measures of small HDL. Fatty acids associations with both the VLDL and remnant and LDL clusters were observed, though the degree of unsaturation, length of fatty acid chain and ratios of fatty acids were associated only with the VLDL cluster, while omega-3 measures were associated with the remnant and LDL cluster. Of note, isoleucine and alpha-1-acid glycoprotein were linked to the VLDL measures cluster, with the later also showing bidirectional associations with the larger HDL measures.

### Adjusting for pleiotropy

Using the MR-Egger approach, as a sensitivity analysis for the effect of pleiotropy in our estimates, resulted in 7074 associations, in accordance with the lower statistical power of the method, with 2224 pairs being bidirectional associations and 2626 in one direction only (Tables S11 and S12). Of the MR-Egger observed associations 6000 were also observed when the standard approach was used. In the children sample, 1719 associations also had evidence for the presence of pleiotropy based on their intercept (Table S13 and S14). MR-Egger analysis of the ALSPAC mothers revealed 176 pairs of bidirectional associations and 305 in one direction, with 137 of these also showing evidence of pleiotropy (Tables S15–S18). Compared to the standard approach 521 pairs of metabolites also had evidence of association in the MR-Egger analysis. In total, the mothers sample provided replication for 486 associations seen in the children.

### Examples

**Fig. 3:**
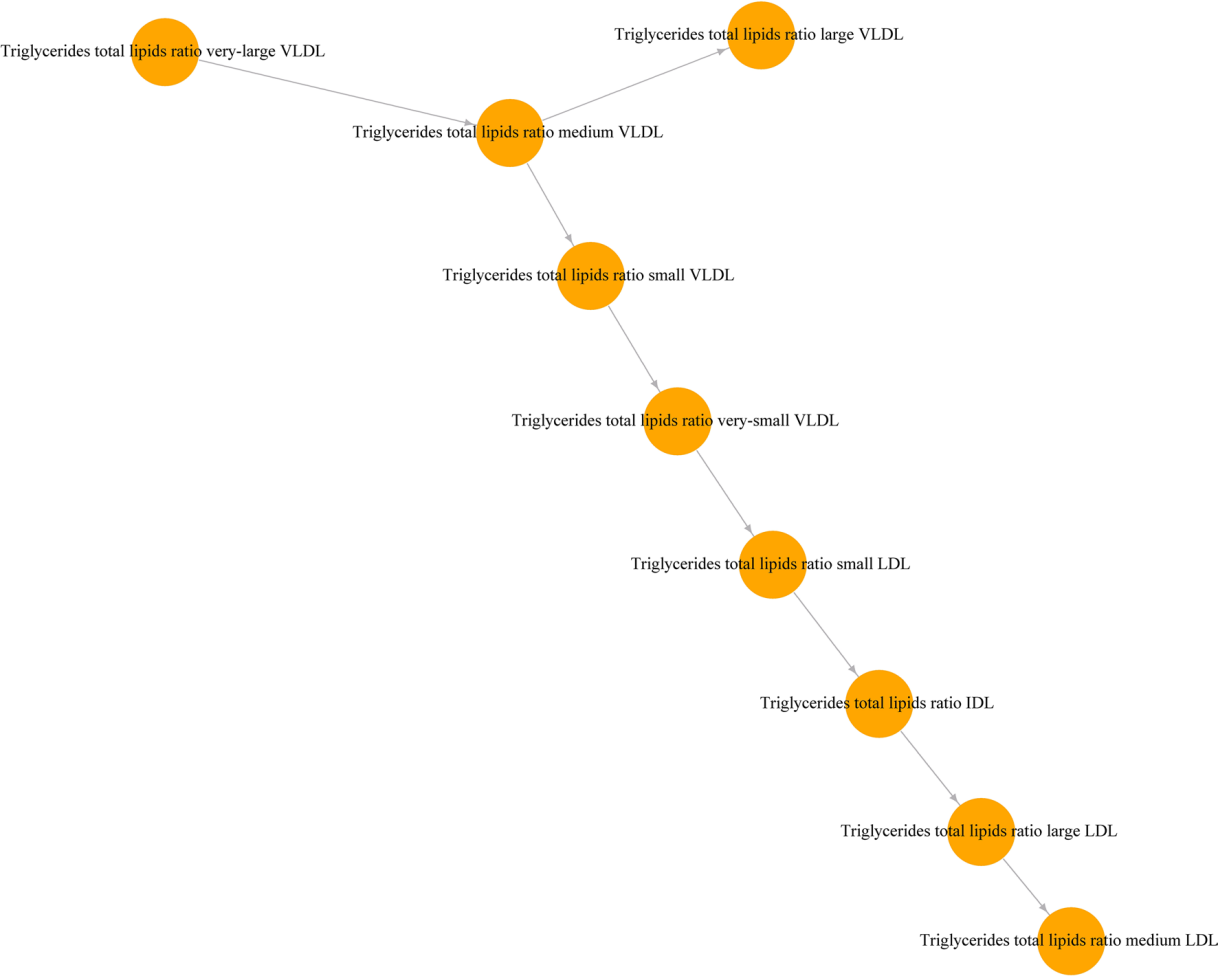
A one directional routed tree representing the strongest (smaller *p*-value) associations between the nine measures of triglyceride concentration in lipoprotein particles

**Fig. 4:**
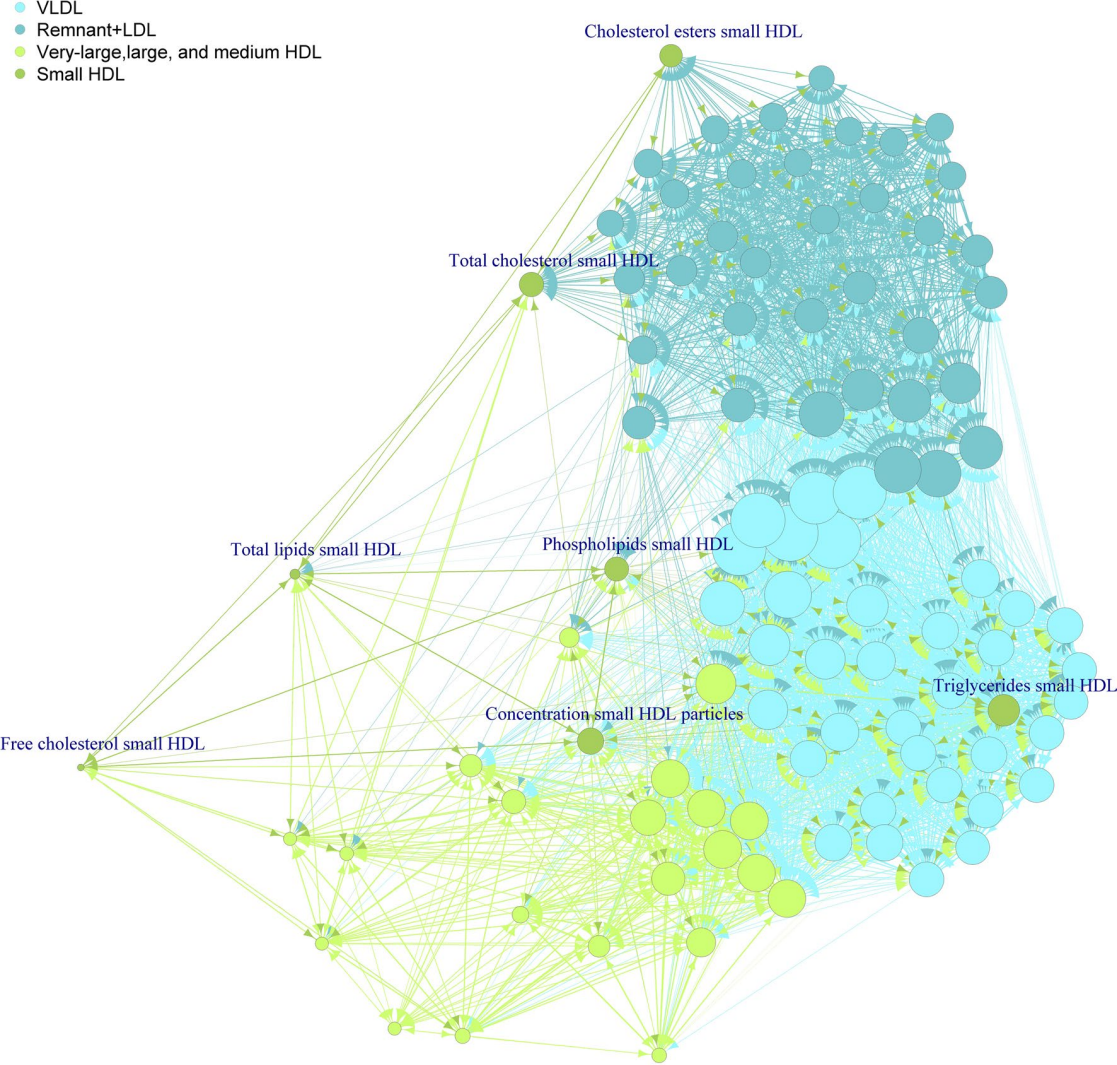
A network of MR replicated associations highlighting the relationships of small HDL measures with other lipoprotein subfractions measures. The size of the node is proportional to the number of connections to the node. The log *p*-value was used as weights for the edges of the network

To demonstrate the potential use of the results in trying to infer the mechanistic relationships between the metabolic measures, we focused on two examples. VLDL is the main transport form of endogenous triglycerides in the body. It is produced in the hepatocytes and released into circulation progressively losing its triglyceride content to give rise to remnant VLDL, IDL and LDL particles of smaller sizes (Marshall et al., 2012). Using the nine measures for the triglycerides to total lipids ratio in VLDL (except chylomicrons and extremely large VLDL which can carry triglycerides from diet), IDL and LDL lipoproteins, a very-large VLDL one directional routed tree (see methods) was constructed, recreating the process of lipoproteins metabolism relatively accurately (Fig. 3). The second example focused on the relationships of the small HDL measures with the rest of the lipoproteins measures (Fig. 4). The triglyceride content of small HDL particles vertex was located within the VLDL measures cluster, while total and esterified cholesterol were closely related with the remnant and LDL cluster, which were the main cholesterol carrying particles. Small HDL total lipids and free cholesterol showed a small number of connections and were situated away from other clusters. The phospholipids in the small HDL vertex had an equal distance from other clusters. Finally, the concentration of small HDL particles was mostly associated and located close to the area of the larger HDL particle measures.

## Discussion

Using two samples of mothers and young participants from a European population cohort and data from a targeted metabolomics platform, predominately of lipoproteins, I illustrate the use of popular epidemiological approaches to assess the relationship between metabolic measures. The results show the presence of strong correlations between the lipoproteins and other measures, at both the phenotypic and genetic levels, and a wide range of causal effects between them, including bidirectional associations. Three main causal clusters of lipoprotein relationships were evident in the results. Heterogeneity of links between HDL lipoprotein particles of different sizes was also observed. Additional insights were obtained for the relationships between CLA, glycolysis markers, and glycoproteins with lipids subfractions.

As the metabolomics platform used is focused mainly on lipoproteins measures, the large number of phenotypic correlations is of no surprise, though similar levels of correlation should be expected within most metabolomics datasets. Except the more obvious reasons of correlations between the metabolic measures, such as the mathematical relationship between them, were ratios are considered, and their proximity in biological processes, more complex mechanisms such as chemical equilibrium, mass conservation, confounding from other internal or external modifiers, and global perturbations due to the conditions of the sample measurement, can also contribute to the correlations observed between metabolites (Camacho et al., 2005; Steuer, 2006).

In the present study, measures of the triglyceride rich VLDL particles made up a distinct group, while the products of their remodelling, triglyceride poor and depleted particles of very small VLDL, IDL and LDL, formed another closely correlated group. These phenotypic correlation features were also present when the genetic correlations were considered, while the MR analysis provided further evidence for both bi-directional and one-directional effects within the two clusters, but very few associations between the clusters. A study on the metabolic profile of statins and the rs12916 *HMGCR* gene polymorphism, identified that disruption of the mevalonate pathway, in addition to the expected LDL lowering effect, also produces large changes on the very-small VLDL and IDL concentration measures and only modest changes on the larger VLDL particles (Würtz et al., 2016b). These results fit very well with the idea of two distinct clusters of associations and the two-way causal effects observed.

The correlations between HDL subfractions were also arranged in two blocks, one for larger and medium and one for small HDL particles. The sub-speciation of HDL to a number of distinct proteins and lipids combinations sharing a similar density has been previously suggested based on proteomics analysis (Davidson et al., 2009) and has been used to explain the large number of properties associated with HDL (Rosenson et al., 2012). The majority of causal associations were between and within the measures of very large and large HDL particles, suggesting that these are quite different from the smaller HDL particles. Interestingly, triglycerides in small and medium, but not large and very large, HDL particles were causally associated with the larger VLDL particles measures and only the smallest HDL particles, corresponding to HDL3, showed evidence of causal effects with the triglyceride depleted, cholesterol rich, low density lipoprotein particles. The observed relationships correspond well to our current understanding of the molecular exchanges taking place during lipoproteins metabolism, with triglycerides moving from VLDL particles to HDL particles, with esterified cholesterol following the opposite direction through the action of the cholesteryl ester transfer protein (Marshall et al., 2016), though now we can provide further information on the size of particles involved.

Other interesting relationships included the associations of conjugated linoleic acid (CLA). CLA is a popular dietary supplement associated with a number of suggested beneficial effects on common diseases and BMI, while recent studies correlated CLA with a decrease of LDL (Derakhshande-Rishehri et al., 2015) and HDL (Kim et al., 2016). We did not find any evidence for a causal effect of CLA on either LDL- or HDL-cholesterol concentrations in the present study. In contrast, we found positive bidirectional associations of CLA with measures of esterified and total cholesterol, as well as triglycerides, in large and very large VLDL particles.

No causal associations between glycolysis markers and lipids were observed. Previously, insulin has been implicated in lipogenesis and VLDL production (Brown & Gibbons, 2001) but a second study looking at the effect of glucose metabolism on the transcriptional regulation of genes involved in VLDL assembly and secretion, did not find any major effects (Morral et al., 2007). The current results do not support the existence of a causal effect between glucose and VLDL concentration and composition measures. Similarly, Citrate, a popular additive to foods and an intermediate product of the Krebs cycle, has been described as a “fundamental precursor” for the endogenous production of cholesterol (Leandro et al., 2016). The observed results do not support any causal associations between plasma measured citrate and lipoprotein measures or glucose.

Evidence for the causal effect of Glycoprotein acetyls, mainly a1-acid glycoprotein (AGP), on large and medium VLDL concentration measures and particle diameter were observed. AGP is an acute-phase protein believed to be involved in a wide range of biological processes, including immuno-modulation, drug compound transport, maintaining capillary function, sphingolipid biosynthesis and glucose and insulin metabolism (Luo et al., 2015). AGP has been suggested as a marker for all-cause mortality (Fischer et al., 2014). The complete function of the protein and how it can interact with VLDL concentration is not known, but our results suggest a role in lipoprotein metabolism that has not previously been identified.

Two examples for the use of the results towards understanding and mapping metabolic networks have been illustrated. The first example looking at the metabolism of triglyceride rich VLDL fits almost perfectly with the current understanding of the process (Marshall et al., 2016). The second example is mapping the characteristics of the small HDL particles in relation to other lipoproteins. According to our results, most of the cholesterol exchange, in the form of esterified cholesterol, is taking place between small HDL and remnant and LDL particles. In contrast, the node of triglyceride concentration of the small HDL particles is embedder within the VLDL cluster of measures. Both features of the network are well established (Marshall et al., 2016). Phospholipids and free cholesterol in small HDL particles are believed to be obtained by the interaction of the early HDL particle with cell membranes (Gurr et al., 2016). This can explain the lack of associations seen with the free cholesterol measure, but the associations with both the VLDL and the remnant and LDL clusters of measures suggests the existence of additional mechanisms.

A number of limitations are evident in this work. The discovery sample was of unfasted children aged 7 with 51.5% of them boys, while the replication sample was of partially related adult fasted women with a mean age of 47.9. This means that the replication sample could confirm the common observed associations, but false positives are indistinguishable from changes due to age and sex. The selection of SNPs to be used as instruments were from the same sample where the MR was performed. This can introduce bias in the analysis resulting in the identification of associations that are not causal (Taylor et al., 2014), but the use of replication and previous biological evidence, suggest that the main associations observed are indeed causal. Finally, we treat each metabolic measure as an independent variable without modelling their potential interactions which will require the use of pathway analysis and latent variables (Burgess et al., 2015), but this will shift the focus of the analysis from hypothesis generating to modelling mediation between a small number of preselected measures.

Focus areas for the use of metabolomics are the understanding and reconstruction of the molecular processes underpinning health and disease and the identification of new risk factors. Genetic variation can be used to provide the direction of effects in a correlation network and disentangle the flow of information in such systems. Here we used measures from a targeted metabolomics platform to explore the correlations between the metabolic measures in the context of common underlying mechanisms with the help of genetic variants. The results of this work provide evidence for, or against, a number of interesting phenotypic associations between the lipoprotein measures and other metabolites and illustrate the challenges and potential uses of this kind of approaches in metabolomics data.

## Supplementary Information

Below is the link to the electronic supplementary material.

Figure S1: Heatmap of Pearson’s correlation coefficients for the pairwise comparison between the metabolic measures in children, below the diagonal, and their respective statistical significance, above the diagonal. The four high correlation clusters are evident. Supplementary file5 (JPEG 12347 kb)

Figure S2:Heatmap of Pearson’s correlation coefficients for the pairwise comparison between the metabolic measures in mothers, below the diagonal, and their respective statistical significance, above the diagonal. The same clusters as before can be seen but the associations are stronger between the clusters. Supplementary file5 (JPEG 12326 kb)

Figure S3: Heatmap of genetic correlations for the pairwise comparison between the metabolic measures in children, below the diagonal, and their respective statistical significance, above the diagonal. The main clusters observed in the phenotypic data are still visible. Supplementary file5 (JPEG 12725 kb)

Figure S4: Heatmap of genetic correlations for the pairwise comparison between the metabolic measures in mothers, below the diagonal, and their respective statistical significance, above the diagonal. The main clusters observed in the phenotypic data are still visible. Supplementary file5 (JPEG 12730 kb)

Supplementary file5 (XLS 15053 kb)
