# Supplementary figures and images for: Systems epidemiology of metabolomics measures reveals new relationships between lipoproteins and other small molecules
Source: Metabolomics. 2021 Dec 16;18(1):1. doi: 10.1007/s11306-021-01856-6 (PMC8683390; doi:10.1007/s11306-021-01856-6)

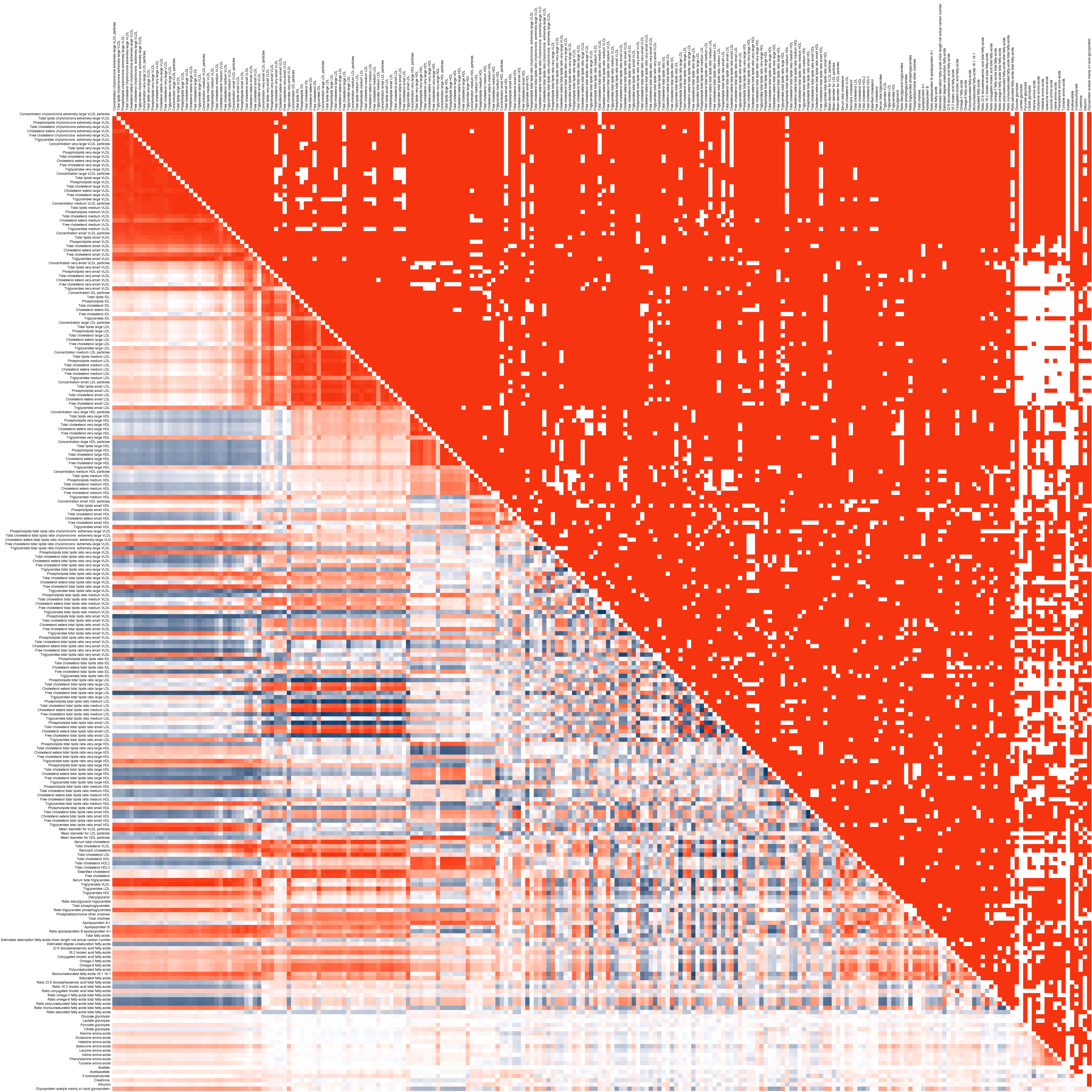

Supplement: Supplementary file 1 — Figure S1: Heatmap of Pearson’s correlation coefficients for the pairwise comparison between the metabolic measures in children, below the diagonal, and their respective statistical significance, above the diagonal. The four high correlation clusters are evident. Supplementary file5 (JPEG 12347 kb) [file 11306_2021_1856_MOESM1_ESM.jpeg]

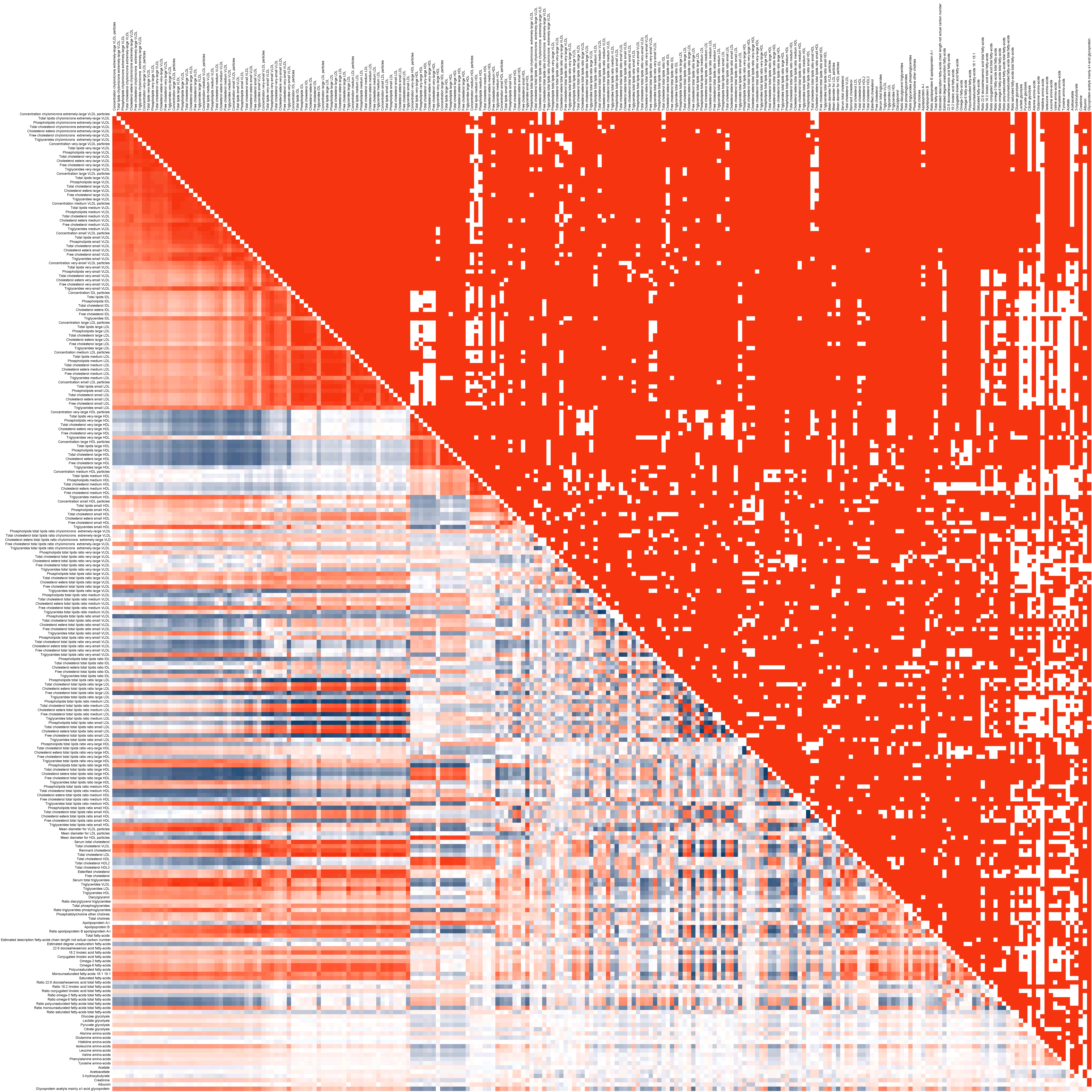

Supplement: Supplementary file 2 — Figure S2:Heatmap of Pearson’s correlation coefficients for the pairwise comparison between the metabolic measures in mothers, below the diagonal, and their respective statistical significance, above the diagonal. The same clusters as before can be seen but the associations are stronger between the clusters. Supplementary file5 (JPEG 12326 kb) [file 11306_2021_1856_MOESM2_ESM.jpeg]

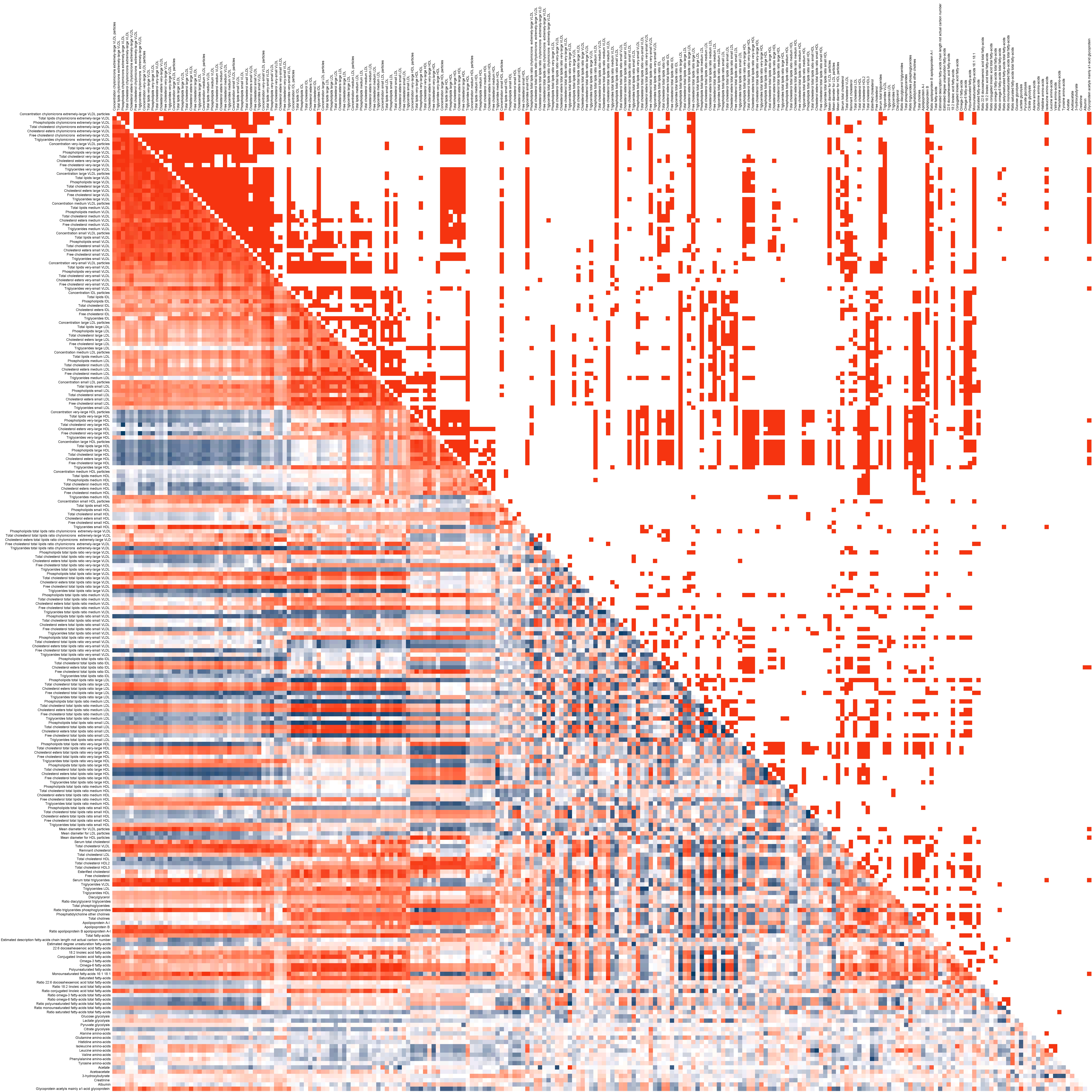

Supplement: Supplementary file 3 — Figure S3: Heatmap of genetic correlations for the pairwise comparison between the metabolic measures in children, below the diagonal, and their respective statistical significance, above the diagonal. The main clusters observed in the phenotypic data are still visible. Supplementary file5 (JPEG 12725 kb) [file 11306_2021_1856_MOESM3_ESM.jpeg]

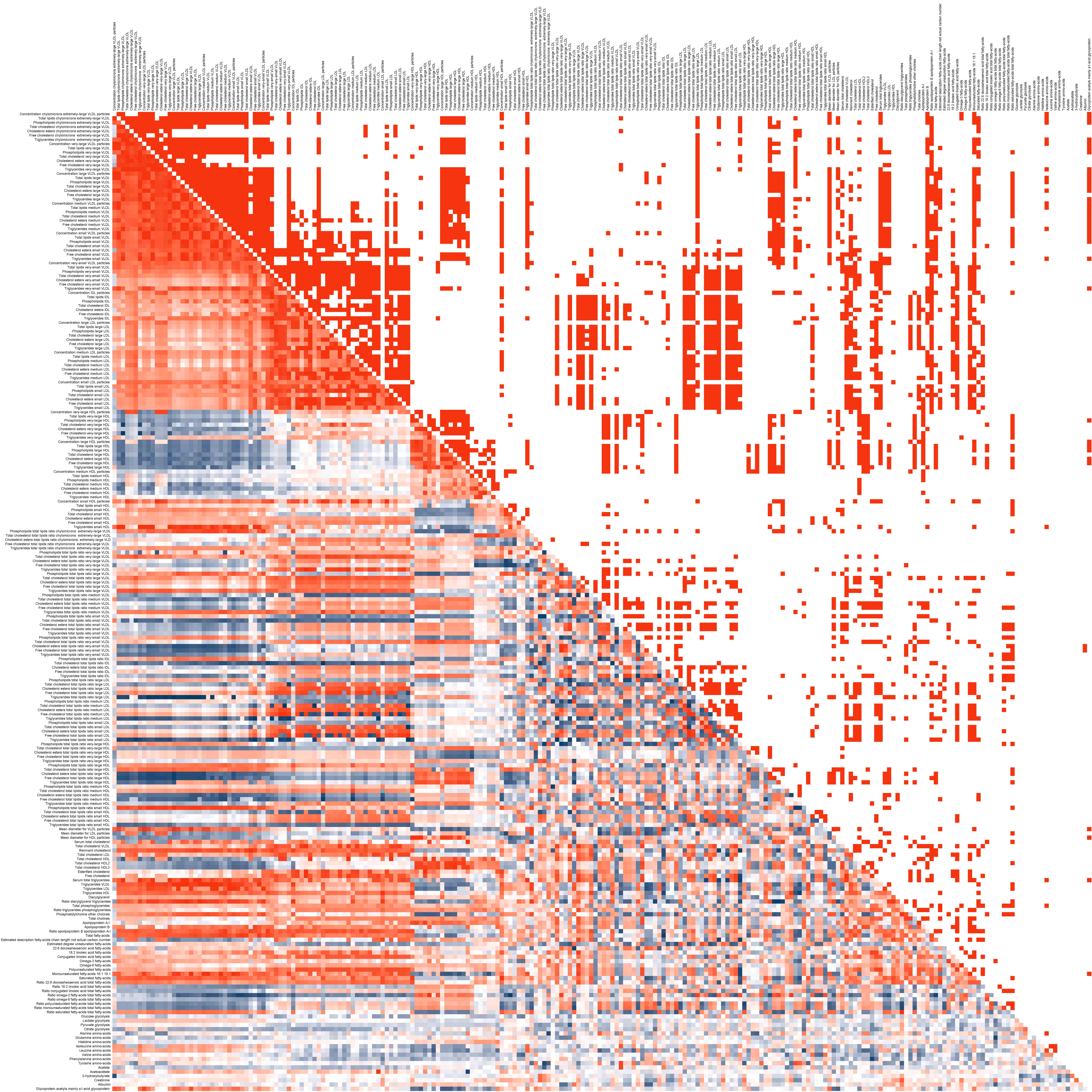

Supplement: Supplementary file 4 — Figure S4: Heatmap of genetic correlations for the pairwise comparison between the metabolic measures in mothers, below the diagonal, and their respective statistical significance, above the diagonal. The main clusters observed in the phenotypic data are still visible. Supplementary file5 (JPEG 12730 kb) [file 11306_2021_1856_MOESM4_ESM.jpeg]
